# Phylogenomic exploration of the relationships between strains of *Mycobacterium avium* subspecies *paratuberculosis*

**DOI:** 10.1186/s12864-015-2234-5

**Published:** 2016-01-26

**Authors:** Josephine M. Bryant, Virginie C. Thibault, David G. E. Smith, Joyce McLuckie, Ian Heron, Iker A. Sevilla, Franck Biet, Simon R. Harris, Duncan J. Maskell, Stephen D. Bentley, Julian Parkhill, Karen Stevenson

**Affiliations:** Wellcome Trust Sanger Institute, Genome Campus, Cambridge, UK; Division of Infection and Immunity, University College London, London, UK; Moredun Research Institute, Pentlands Science Park, Penicuik, EH26 0PZ UK; Institute of Infection, Immunity & Inflammation, University of Glasgow, Glasgow, G12 8QQ UK; Neiker-tecnalia, Dpto. de Producción y Sanidad Animal, Berreaga 1, 48160 Derio, Bizkaia Spain; INRA, UMR1282, Infectiologie Santé Publique (ISP-311), F-37380 Nouzilly, France; Department of Veterinary Medicine, University of Cambridge, Cambridge, UK

**Keywords:** *Mycobacterium avium* subspecies *paratuberculosis*, Johne’s disease, Genome wide sequencing, Phylogenomics, Genotyping

## Abstract

**Background:**

*Mycobacterium avium* subspecies *paratuberculosis* (*Map*) is an infectious enteric pathogen that causes Johne’s disease in livestock. Determining genetic diversity is prerequisite to understanding the epidemiology and biology of *Map*. We performed the first whole genome sequencing (WGS) of 141 global *Map* isolates that encompass the main molecular strain types currently reported. We investigated the phylogeny of the *Map* strains, the diversity of the genome and the limitations of commonly used genotyping methods.

**Results:**

Single nucleotide polymorphism (SNP) and phylogenetic analyses confirmed two major lineages concordant with the former Type S and Type C designations. The Type I and Type III strain groups are subtypes of Type S, and Type B strains are a subtype of Type C and not restricted to *Bison* species.

We found that the genome-wide SNPs detected provided greater resolution between isolates than currently employed genotyping methods. Furthermore, the SNP used for IS*1311* typing is not informative, as it is likely to have occurred after Type S and C strains diverged and does not assign all strains to the correct lineage. Mycobacterial Interspersed Repetitive Unit-Variable Number Tandem Repeat (MIRU-VNTR) differentiates Type S from Type C but provides limited resolution between isolates within these lineages and the polymorphisms detected do not necessarily accurately reflect the phylogenetic relationships between strains.

WGS of passaged strains and coalescent analysis of the collection revealed a very high level of genetic stability, with the substitution rate estimated to be less than 0.5 SNPs per genome per year.

**Conclusions:**

This study clarifies the phylogenetic relationships between the previously described *Map* strain groups, and highlights the limitations of current genotyping techniques. *Map* isolates exhibit restricted genetic diversity and a substitution rate consistent with a monomorphic pathogen. WGS provides the ultimate level of resolution for differentiation between strains. However, WGS alone will not be sufficient for tracing and tracking *Map* infections, yet importantly it can provide a phylogenetic context for affirming epidemiological connections.

**Electronic supplementary material:**

The online version of this article (doi:10.1186/s12864-015-2234-5) contains supplementary material, which is available to authorized users.

## Background

*Mycobacterium avium* subspecies *paratuberculosis* (*Map*) is an enteric pathogen causing Johne’s disease, which is responsible for considerable economic losses to the livestock and associated industries on a global scale [1]. *Map* can infect a broad range of host species [2–4], but clinical disease is reported only in ruminants [5], camelids [6, 7], rabbits [8] and hares [9]. *Map* has been detected in humans in a subset of patients with Crohn’s disease [10]. Although the zoonotic potential of *Map* remains a controversial issue, its presence in the food chain is an important consideration for the food industry and there is a drive towards controlling the organism on the farm.

Understanding the genetic diversity of *Map* is important for both epidemiological and biological reasons and will inform the development of improved diagnostics and effective vaccines for controlling disease. However, like the related human pathogens *Mycobacterium tuberculosis* and *Mycobacterium leprae*, *Map* is genetically monomorphic [11] so presents a challenge to genotyping. Over the years, various molecular typing techniques have been used to differentiate between *Map* isolates (for reviews see [12, 13]). In 1990, Collins et al. [14] described two major groups of strains, which appeared to correlate with the host of origin and were designated “Sheep-type” (Type S) or “Cattle-type” (Type C). However, as strain typing was more widely applied, it became apparent that the correlation between strain type and host species was not absolute and it was not always clear when the ‘sheep’ or ‘cattle’ designation referred to the strain type or the host provenance. To avoid confusion, it was proposed that these strain types be referred to as Type I (Type S) or Type II (Type C) [15], although both designations are still in use. These two major strain groups can be differentiated based on their genotype, growth characteristics and pathogenesis [13, 16].

Other strain groups have been identified. A group of strains designated “Type III” has been described [17–19] and was suggested to represent an evolutionary intermediate between the two major strain groups. ‘Bison’ (Type B) strains comprise another group of strains. These strains were first isolated from bison (*Bison bison*) in Montana, USA and although molecular analysis characterized them as Type C, the unusual growth requirements of the isolates and clinical presentation in the infected animals suggested that they may be different from the strains commonly isolated from cattle [20]. Initially these strains were differentiated on the basis of the number of copies with a C or a T at base position 223 in the insertion sequence IS*1311* [20]. Subsequent genotyping of more isolates from bison demonstrated that isolates obtained from bison in India were different from those from US bison and these have been referred to as ‘Indian bison type’ [21]. Sohal et al. [22] recently identified a unique TG deletion at base pair positions 64 and 65 of IS*1311* at locus 2 in the Indian bison type strains.

Whilst these different strain groups have been defined using different typing procedures, the phylogenetic relationships between them have not been elucidated. In this study we undertake the first whole genome sequencing (WGS) study using a comprehensive international panel of strains to determine the evolution, population structure and phylogeography of *Map*. We undertake single nucleotide polymorphism (SNP) analysis of a panel of 141 *Map* isolates representing 17 countries, nine host species and all of the strain groups described above to determine the extent of genetic diversity and phylogenetic relationships between the strains. In addition, we assess the performance of current typing techniques compared to the ultimate level of resolution obtained with WGS to assess their utility for epidemiological studies, surveillance and tracing of infections. Finally we investigate the stability of the genome following *in vitro* passage and provide an estimate for the *Map* mutation substitution rate.

## Methods

### Panel of strains

For this study, a panel of 141 *Map* isolates was carefully selected to maximize genetic diversity and include representative isolates for all strain groups identified to date. Details of the isolates comprising the panel are given in Additional file 1: Table S1. To maximize genetic diversity, isolates were chosen with different multiplex pulsed field gel electrophoresis (PFGE) profiles (total of 68) and selected from different geographical regions representing 17 countries (Argentina, Canada, Czech Republic, Faroe Islands, France, Germany, Greece, India, Ireland, Italy, The Netherlands, New Zealand, Norway, Spain, United Kingdom, United States of America and Venezuela). The isolates were from cattle, sheep, goats, deer (unspecified species), bison (*Bison bison*), buffalo (*Bubalis bubalis*), moufflon (*Ovis musimon*) and humans with Crohn’s disease. Four vaccine strains and an environmental isolate from water were also included. In total, the panel comprised 20 Type S isolates, (of which 14 were Type I and six Type III), eight Type B isolates and the remaining 112 isolates were Type C. Twelve strains with identical genotypes as determined by a combination of PFGE and Mycobacterial Interspersed Repetitive Unit-Variable Number Tandem Repeat (MIRU-VNTR) typing were included to investigate the discriminatory power of WGS and multiple passages of two field strains and the reference strain *MapK10* were included to investigate genome stability. All isolates were positive for IS*900*. PFGE was performed according to the standardized protocol published by Biet et al. [23]. MIRU-VNTR analysis was performed as described by Thibault et al. [24] and the profile number assigned by INRA and published in an online database [25]. The presence of LSP^A^20 (for Type C strains) and LSP^A^4-II (for Type S strains) was determined by PCR using primers reported by Semret et al. [26] and conditions described by Biet et al. [23]. Type I and III strains were assigned according to the SNP analysis of the *gyr*A and *gyr*B genes as described by Castellanos et al. [19] or according to their PFGE profile [15, 17]. SNP analysis of IS*1311* by PCR restriction enzyme analysis to determine types S, C and B was undertaken as described by Whittington et al. [20]. Two other isolates representing members of the *Mycobacterium avium* complex (*Mycobacterium avium* subspecies *avium* [*Maa*] and *Mycobacterium avium* subspecies *silvaticum* [*Mas*]) were included in the panel for WGS to place the *Map* strains in a wider mycobacterial context.

### Culture of isolates

Isolates were received from participating laboratories growing on a variety of media and were subsequently propagated on 7H11+ agar (Middlebrook 7H11 supplemented with 20 % [vol/vol] heat-inactivated newborn calf serum, 2.5 % [vol/vol] glycerol, 2 mM asparagine, 10 % [vol/vol] Middlebrook oleic acid-albumin-dextrose-catalase [OADC] enrichment medium [Becton Dickinson, Oxford, Oxfordshire, United Kingdom], Selectatabs [code MS 24; MAST Laboratories Ltd., Merseyside, United Kingdom], and 2 μg ml^−1^ mycobactin J [Allied Monitor, Fayette, Mo.]). For preparation of DNA, the isolates were subcultured in Middlebrook 7H9 broth supplemented with 0.2 % (vol/vol) glycerol, 0.05 % (vol/vol) Tween 80 and 2 μg ml^−1^ mycobactin J and stirred during incubation at 37 °C.

For investigating genome stability, the two field strains M21/02 and JD143 and *Map* K10 were subcultured on 7H11+ agar every 6–12 weeks. The passage number was noted and glycerol stocks prepared and archived at −80 °C for each passage.

### Preparation of DNA

Mycobacteria were harvested in early to mid log phase of growth and the cells were pelleted at room temperature for 5 min at 14,000 g. Pellets were re-suspended in ATL buffer (Qiagen DNeasy^®^ Blood & Tissue Kit) and the samples were transferred to Lysing Matrix B tubes (0.1 mm silica spheres). Samples were homogenised in a FastPrep™ FP120 cell disruptor at 3 × 20s, Speed 6, followed by centrifugation at 14,000 g for 5 min. Supernatants were transferred to fresh sterile micro centrifuge tubes and Proteinase K (Qiagen DNeasy^®^ Kit) was added followed by incubation overnight at 56 °C. DNA was extracted using DNeasy^®^ Kit (Qiagen) according to the manufacturer’s protocol.

### Genome sequencing

DNA library preparation was carried out by the DNA sequencing teams at the Wellcome Trust Sanger Institute. Multiplexed libraries were sequenced in batches of 12 on the Illumina Genome Analyzer (GAIIx platform) platform to produce 75 bp paired end reads.

### SNP analyses and phylogenomics

The sequencing data were mapped against the *Map* K10 reference [27] using SMALT (http://sourceforge.net/projects/smalt/) with default parameters. Consensus variants were called using SAMtools and bcftools [28] using filters designed to keep false positives to a minimum, which include a minimum base quality of 50, a minimum mapping quality of 30, support from at least 4 reads (2 forward and 2 reverse) and an absence of heterozygosity. Previously these filters have been shown to keep the false positive rate to lower than 1 SNP per genome [29]. Variants that passed these filters were used to build a maximum likelihood tree using RAxML v. 7.0.4 [30] with 100 bootstrap replicates.

The BEAST package (v1.7.5), a program for Bayesian Markov chain Monte Carlo (MCMC) analysis of genetic sequences, was used to estimate substitution rates [31]. Input XML files were created using BEAUTi [30] from the whole genome SNP-alignment and the associated dates of isolation for each isolate. For all analyses, three independent MCMC chains of 100 million states were run using a GTR evolutionary model and a variety of combinations of clock and population size models. Tracer (v1.5) [31] was used to assess convergence (after an initial burn-in period of 10 %), agreement between the three runs and that all effective sample size (ESS) values were greater than 200.

*IS1311* typing was carried out *in silico*, using the raw sequencing data. All reads were mapped to a representative sequence (locus tag MAP4_RS00055 in reference accession NC_021200.1) using the mapping approach described above, and the proportion of reads mapping to the IS*1311* element with a T or C at position 223 was counted. The presence of the “TG” deletion [22] was assessed by parsing of the bcf file and manual inspection. The position of the IS*1311* elements in the genome of each isolate was predicted by first identifying the corresponding forward/reverse read of the reads identified as matching IS*1311* (from above). These were then mapped to the *Map* K10 reference [27] using SMALT with repeat mapping switched off. The specific position of these reads indicates the position of the IS*1311* element.

Publically available Camelid genome sequences [6] were incorporated into the phylogenetic tree by simulating short reads from the *de novo* assembly, and following the variant calling procedure described above. To do this an in-house script was used to create 75 bp paired reads, assuming a 200 bp insert size in a moving window procedure for every 3 bp, resulting in an approximate depth of 25×. These simulated reads will not have sequencing errors like normal short-reads, however this approach relies on the accuracy of the original *de novo* assembly so results should be treated with caution.

### Availability of supporting data section

Raw sequence data supporting the results of this article are deposited in the European Nucleotide Archive (ENA) under accession PRJEB2204.

## Results and discussion

### Phylogenetic classification of *Map* isolates

**Fig. 1:**
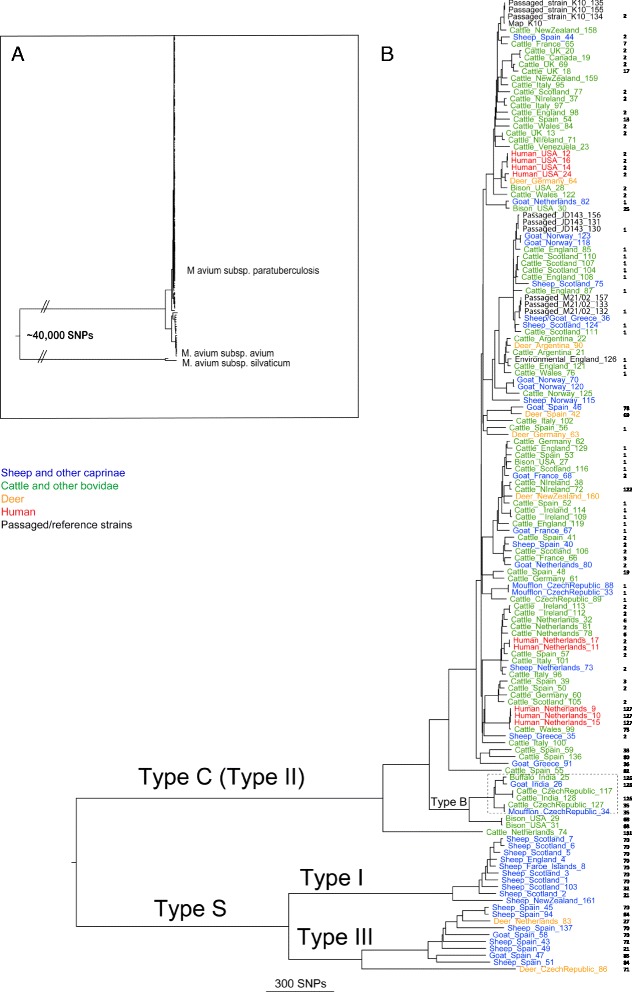
Whole genome SNP-based phylogenetic tree of strains included in this study. **a** Phylogenetic tree of *Map* and non-*Map* strains built using FastTree [52]. *Map* strains form a single clade and are separated from other mycobacterial species by at least 40,000 SNPs (branches shortened for illustrative purposes). **b** Maximum likelihood phylogenetic tree of *Map* strains sequenced as part of this study. The tree was created using RAxML [30], and is based on SNPs identified through mapping to *Map*-K10 as described in the text. Branches are annotated with the host, country of origin and isolate MAPMRI numbers. Numbers in black to the far right represent the INMV profiles. Previously described lineages are labeled. The dashed box represents strains designated as the Indian bison type. Bootstrap values are shown in Additional file 2: Figure S1

WGS data were obtained for 141 isolates. The sequences were mapped to the corrected and annotated reference *Map* strain K10 [27] to identify SNPs. A maximum likelihood phylogenetic tree based on SNPs detected in non-repetitive regions of the genome is depicted in Fig. 1 and Additional file 2: Figure S1. *Maa* and *Mas* were included to place the *Map* strains in a wider mycobacterial context as shown in the inset in the figure. There were more than 40,000 SNPs between the *Map* isolates and these other members of the *Mycobacterium avium* complex. SNP distances between the major strain groups are shown in Table 1. The phylogenetic analysis confirmed the division of *Map* isolates into two major lineages, concordant with the previous Type S and Type C designations. We propose that this nomenclature remains for historical reasons but re-emphasise the fact that it does not necessarily reflect host provenance. The Type S clade included isolates from sheep, goats and deer and the Type C clade comprised isolates from cattle, sheep, goats, deer, moufflon, bison, buffalo and humans. Type S strains are perceived to have a host preference for sheep and goats but this observation has been largely based on the low frequency of isolation of Type S strains from other species and could reflect a lack of interaction between the host species rather than a true adaptation to specific host populations. A recent study by Verdugo et al. [32] reported that Type S strains are more frequent in New Zealand beef cattle than Type C strains where these species are frequently grazed together. Type C strains are isolated from a broad range of hosts and do not appear to have a host preference. The evidence for interspecies transmission is compelling, but the relative risk of transmission of the different strain types between host species cannot be determined with our data. It has been reported that the risk of natural transmission of Type S strains from sheep and goats to cattle is low and occurs only when susceptible animals are exposed to high doses [33].

**Table 1:** Distance matrix plot showing the number of SNPs present between selected strain groups

	Type S	Type S(I)	Type S(III)	Type C
Type S				2360
Type S(I)			1051	2684
Type S(III)		1051		3087
Type C	2360	2684	3087	
Type B	2565	2889	3292	264

The phylogenetic analysis clarified the relationship between the previously described Type I and Type III strains. These strain groups are distinct sublineages of Type S, and Type III is not intermediate between the Type S and Type C strains as previously suggested. The Type I strains comprise nine of ten pigmented strains but not all strains in this group are pigmented. A New Zealand ovine isolate, MAPMRI161, is not pigmented but is closely related to the Type I group and a Spanish ovine isolate, MAPMRI051, in the Type III group is pigmented. We failed to identify any SNPs, insertions, deletions or the presence or absence of any single gene that could be exclusively associated with pigmented strains. This suggests that the underlying genetic basis for changes in this phenotype is likely to be complex and multi-factorial.

Phylogenetic analysis confirmed that the Type B strains are a subtype of Type C and not restricted to *Bison* species. The USA Type B isolates could be differentiated from ‘Indian bison type’ by 255 SNPs and also by PFGE data from this project and data reported previously [21].

The human isolates from Crohn’s disease patients did not comprise a distinct phylogenetic group, consistent with previous observations [34, 35] but were all Type C strains. There were no SNPs, insertions or deletions found to be exclusive to these isolates. However, although the human isolates did not comprise a single group, we did observe that human isolates from the same country were often closely related (Fig. 1 and Additional file 3: Figure S2). We know of no epidemiological connections between the isolates except for MAPMRI009 and MAPMRI010, which were isolated from the same patient employing different techniques. Furthermore, the human isolates appeared to be closely related to *Map* isolates from sheep and cattle from the same country, suggesting livestock as a possible source of transmission.

Since this work was initiated, further *Map* isolates have been sequenced and their genome sequences made available in the public databases. These include the Camelid strains JQ5 and JQ6 [6], S5 Indian bison-type [36], USA Type S isolate S397 [37], Australian ovine isolate CLIJ361 [34], two Australian bovine isolates CLIJ623 and CLIJ644 [34], Australian human isolates Pt139, Pt144, Pt145, Pt146, Pt154, Pt155 and Pt164 [34], USA caprine isolate JTC1285 and an isolate from an oryx JTC1281 [35]. To identify the phylogenetic relationships between these strains and those in our panel, we included these strains in our analysis and the resulting phylogenetic tree is depicted in Additional file 3: Figure S2. It is clear from the analysis that the available camelid strains represent another subtype of Type S. The S5 Indian bison-type strain clustered with the other non-USA Type B strains confirming the existence of an Indian bison-type group. The USA S397 isolate was most closely related to the Type III isolates originating from Spain whereas the Australian ovine isolate CLIJ361 was most closely related to a New Zealand Type I isolate. The remaining isolates were distributed among the Type C isolates.

In general, there did not appear to be a strong association with geographic location among the isolates in this panel. However, the results are biased in that the isolates were chosen to maximise genetic diversity and have been assembled from a global perspective but with a limited number of isolates from many included countries. WGS of larger numbers of isolates from defined geographic locations may reveal correlations.

### Relationship between IS*1311* genotype and the whole genome phylogeny

**Fig. 2:**
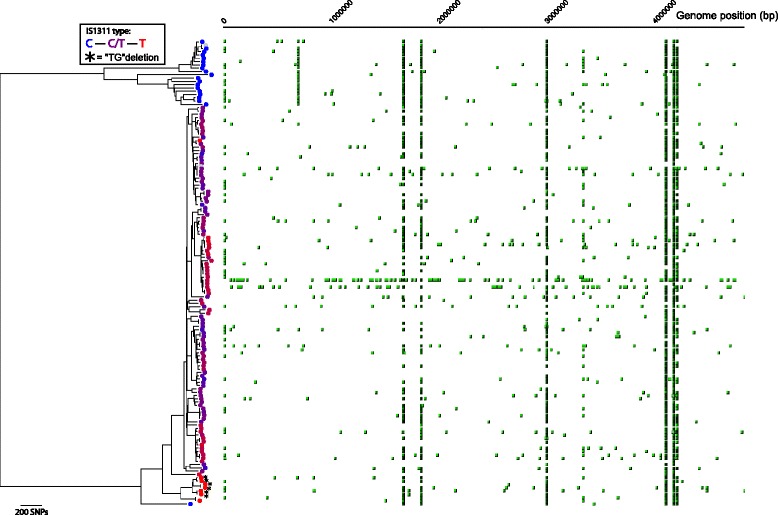
Type and genomic position of IS*1311* element found in the *Map* sequencing data. The proportion of reads mapping to the IS*1311* element with a T or C at position 223 is indicated by the colour gradient of circles displayed on the phylogenetic tree. Asterisks indicate samples where the “TG” deletion was detected [22]. The position of the IS*1311* element in the genome was predicted by identifying paired reads where one read maps to the IS*1311* element and the other does not, and using the mapped position of the latter. This is displayed horizontally with the genome co-ordinates displayed along the top. The colour intensity of the predicted IS*1311* element position represents the relative number of supporting reads detected within the sample, and thus is normalised for differences in depth of coverage between samples

IS*1311* genotyping is used in many laboratories to distinguish between S, C and B-type strains. The strains are differentiated on the basis of the number of copies with a C or a T at base position 223 in the insertion sequence IS*1311* [20]; in this scheme all copies of IS*1311* in B-type strains have a T at position 223 whereas all the Type S strains have a C and the Type C strains have one or more copies with a C or a T at the same position. IS*1311* genotyping of isolate MAPMRI074 identified the isolate as a Type S strain but its position in the phylogenetic tree placed it as a Type C. This anomaly prompted us to look at the distribution of C and T alleles at base pair position 223 for the panel isolates. Reads that mapped to the IS*1311* element were identified, and the proportion of base calls for either a T or C at nucleotide position 223 was counted as shown in Fig. 2. All Type S strains in the panel have a C at this position and all of the Type B strains have a T as previously reported [20]. All Type B strains had evidence of the “TG” deletion at positions 64 and 65 as previously observed [22]. No differences were observed between the USA bison strains MAPMRI029 and MAPMRI031 and the Indian bison-type group using this assay. However, it is clear that differences exist between the other non-Type B, Type C strains with respect to the number of copies of IS*1311* with the T-C allelic variation. The results for some Type C strains not in the Type B cluster suggest that these strains also have the T allele at base position 223 in all copies of the IS*1311* element. As isolate MAPMRI074 was identified as a Type S strain on the basis of an unequivocal result in the IS*1311*-REA and IS*1311* sequence analysis but was clearly a Type C strain confirmed by its positivity for LSP^A^20 and its position within the phylogenetic tree, it suggests that the C to T allelic variation at base pair 223 in IS*1311* occurred after the initial divergence of Type C from Type S strains. This is the first Type C *Map* isolate to be identified with a Type S IS*1311*-REA profile. The isolate did not exhibit any notable phenotypic differences compared with the other Type C isolates with respect to growth characteristics. These results suggest that the IS*1311*-REA may not be the best assay for differentiating between Type S, Type C and Type B. The PCRs for LSP^A^20 and LSP^A^4-II [26] may be the better choice for distinguishing Type C and Type S strains.

### Relationship between MIRU-VNTR genotype and the whole genome phylogeny

MIRU-VNTR genotyping is based on detecting the number of copies of mycobacterial interspersed repetitive units (MIRUs) and variable number tandem repeats (VNTRs) in the genome, which differ between strains. This is probably the most commonly used genotyping method for *Map* as it is easy to perform, requires only a small amount of DNA and the digital sequence profiles can be stored in databases and compared between laboratories [12]. For this study we used the eight MIRU and VNTR loci described by Thibault and coworkers [24]; MIRUs 292 and X3 and VNTRs 25, 47, 3, 7, 10 and 32. Profile numbers (designated INMV numbers) were assigned by INRA and collated in a publically available database [25]. MIRU-VNTR data available for 105 sequenced isolates were mapped onto the phylogenetic SNP-based tree as shown in Fig. 1 and Additional file 4: Figure S3. A total of 30 different INMV profiles were represented among the isolates.

As expected we observed clear differences in the MIRU-VNTR profile between the Type S and Type C lineages as reported previously by Biet et al. [23]. However, within these lineages MIRU-VNTR provided less discriminatory power, where many isolates shared identical profiles. For example, of the 82 MIRU-VNTR typed Type C strains, 27 and 31 had an identical profile defined as INMV1 and INMV2 respectively, which are the most common profiles across Europe [38] and Canada [11]. Simpson’s index of diversity [39] for Type S strains in the panel is 0.7251 and for Type C strains 0.7515.

Our study strongly supports previous reports that MIRU-VNTR genotyping does not accurately reflect phylogeny and that these repeat sequences are subject to homoplasy [11, 40, 41]. Homoplasy is the occurrence of genotypes that are identical by state but not by descent and can arise by various means including convergent and reverse evolution and horizontal gene transfer. Homoplasy was evident in five of 11 INMV types in which more than one isolate per type was sequenced (INMV 1, 2, 3, 21 and 70). Homoplasy among INMV 2 and 3 was also observed by Ahlstrom et al. [11]. INMV types 21 and 70 were found in both the Type I and Type III sublineages. Hence this first study of a global panel of *Map* isolates emphasizes that MIRU-VNTR could provide misleading genetic and epidemiological relationships between strains and any inference of transmission from identical INMV profiles must be made with caution. In contrast only 2 % of SNPs were observed to be homoplasic, further highlighting the strength of the whole genome approach over MIRU-VNTR typing. This is exemplified by the 12 epidemiologically unrelated isolates with identical combined PFGE ([2-1]), MIRU-VNTR (INMV1) profiles, which were included in our study (Additional file 1: Table S1). The phylogenetic analysis showed that these isolates belonged to five different clades within the Type C lineage (Fig. 1 and Additional file 4: Figure S3), demonstrating that SNP analyses outperform MIRU-VNTR even when the latter is combined with other genotyping techniques.

### Genomic changes following continuous passaging *in vitro*

WGS was performed on three passages (P1, P26 and P32 or 37) of strains M21/02, JD143 and *Map*K10 and the sequences compared to determine any changes in the genome between passages. The field strains M21/02 and JD143 were passaged from primary isolation on 7H11+, P1 representing the first subculture. The *Map*K10 strain was obtained from the American Type Culture Collection (ATCC BAA 968) and there are no data regarding how many times this strain had been passaged. Therefore, for this strain P1 represents the first passage in our laboratory. For strain M21/02 a SNP (A → C) was detected at position 2041004 at P26 causing a non synonymous mutation in the MAPK_1803 (*glnE*) gene. This SNP was also detected at P37 but no further changes were observed. For JD143, no differences were observed between P1 and P26 but after 37 passages four mutations were detected; two synonymous SNPs were detected at position 812776 in the MAPK_0714 (*ligA*) gene and at position 4151948 in the MAPK_3716 (*glnQ*) gene and two non synonymous SNPs at position 1488559 within MAPK_1342 (a pseudogene) and at 1687561 in MAPK_1510 (*nadE*). For *Map*K10 an insertion of a G at position 4410158 within MAPK_3949 was detected at P26, which causes a frameshift in *mmpL4_7*. This mutation was also present at P32.

It is interesting that so many of these mutations affect genes involved in glutamine pathways for nitrogen assimilation. Glutamine and glutamate are the two major amino acids that act as cellular nitrogen donors for the synthesis of biomolecules within the cell [42]. In mycobacteria, the assimilation of inorganic nitrogen and its conversion to glutamine and glutamate is carried out by glutamine synthetase whose activity is modulated by GlnE, an adenylyl transferase [43]. NadE is a glutamine-dependent NAD(+) synthetase that obtains ammonia through the hydrolysis of glutamine to glutamate and GlnQ is a glutamine ABC transporter ATP-binding protein. GlnE is essential for growth of *M.tuberculosis* [44] whereas GlnQ is a non-essential gene and is in fact deleted in some clinical *M.tuberculosis* isolates. It is tempting to suggest that the mutations arising in these genes in the two field strains are adaptations to the growth medium. The *Map* field isolates were propagated on 7H11+, which contains ammonium sulphate, L-glutamate, ferric ammonium citrate and L-asparagine as sources of nitrogen. Under these conditions, nitrogen is not a limiting factor and the cells would not need to expend energy acquiring nitrogen. It is known that media constituents can modulate aerobic expression of genes and operons, for example, *devR-devS* [45]. In addition, genomic changes in *Map* potentially arising as a result of long term growth on potato starch medium and Dubos medium with added pyruvate have been reported [46]. Interestingly, Hsu et al*.* [35] reported a SNP in the *glnE* gene in all six *Map* genomes sequenced and postulated that this might be an indication of common evolutionary ancestor with environmental isolates. It is possible that the *glnE* gene may be a hotspot for mutation in the genome allowing for rapid adaptation to niche-specific conditions.

The frameshift mutation within *mmpL4_7* in MapK10 is also interesting. Inactivation of *mmpL* genes typically leads to a change in surface characteristics, which could include altered colony morphology, reduced sliding motility and reduced biofilm formation. These characteristics were not observed on the 7H11+ slopes during this study. The *mmpL* genes are involved in virulence in *M.tuberculosis* [47]. MmpL4 is required for optimal growth of *M.tuberculosis* and survival in mouse lungs [47] and is down regulated during nutrient starvation [48]. Therefore, a mutation in MmpL4_7 potentially could reduce the virulence of the *Map*K10 strain.

### Estimation of the substitution rate in *Map*

**Fig. 3:**
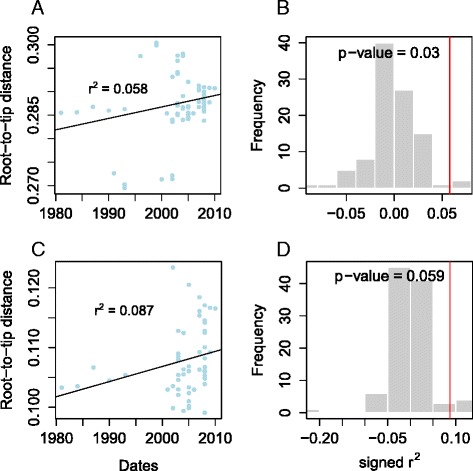
Investigating the presence of a clock-like signal in the data. The presence of a molecular clock in the dataset was assessed by plotting the root to tip distance of isolates in the phylogeny against isolation date [53] for both the dataset as a whole (**a** + **b**) and only Type II isolates (**c** + **d**). Both had evidence of a very weak positive signal, as indicated by a low linear regression correlation coefficient (**a** + **c**). In order to test the significance of these observations, 99 comparison datasets were produced, in which the isolation dates were permuted on the phylogeny. **b** and **d** show histograms of correlation coefficient values from the permuted datasets, with the red line indicating the value from the real data. In both cases the correlation coefficient of the real data is significantly greater than the permuted data at the 0.05 level. Passing this test is a minimal requirement for the application of BEAST analysis (Fig. 4), which assumes the presence of a molecular clock

**Fig. 4:**
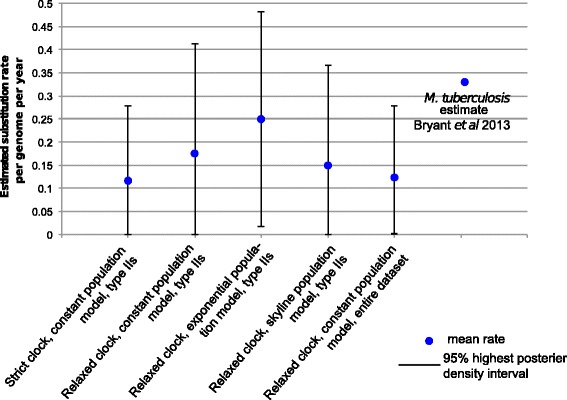
Estimates of substitution rate. Coalescent analyses were implemented using the BEAST package (v1.7.5) [31] as described in the text. This was carried out both on the entire data and the Type C isolates alone, using a variety of population models as shown. The mean value represents the mean estimated substitution rate from three independent runs. The confidence intervals represent the maximum and minimum higher posterior densities obtained. As a comparison, the estimated rates are shown alongside a predicted substitution rate of *M. tuberculosis* in the context of transmission [49]

We investigated the presence of a molecular clock in the dataset by plotting the root to tip distance of every isolate in the phylogeny against the date of their isolation. This was carried out on the dataset as a whole (where the date of isolation was available) as well as on only Type-C isolates, using a variety of different evolutionary and population models. A weak positive signal was detected (Fig. 3a, c), providing weak evidence for the presence of a molecular clock. However, when this signal was compared to those obtained from trees where isolation date was randomized, this relationship was found to be statistically significant for the entire dataset but not Type Cs in isolation (Fig. 3b, d), which provides support for the presence of a molecular clock in this dataset. Coalescent analysis implemented in BEAST was used to estimate the substitution rate in *Map* (Fig. 4). All estimates agreed on a low substitution rate, with an upper limit of less than half a SNP per genome per year. Considering the average mutation rate of 0.3 SNPs per genome per year estimated for *M.tuberculosis* [49] and the longer doubling time of 22–26 h for *Map* [50] compared with 16 h for *M.tuberculosis* in optimal laboratory conditions [51], a lower average mutation rate would be consistent for *Map*

The low sequence diversity and substitution rate for *Map* has implications for epidemiological investigations tracing the sources of infections. Even with the ultimate resolution afforded by WGS, this alone will not be sufficient to track transmission or reconstruct outbreaks. However, constructing phylogenetic trees from SNP data could help to determine if a recent transmission event has occurred. For example, isolates that represent recent transmission events would be expected to be located adjacent to one another on the tree and share a common ancestor. Regardless of WGS data, epidemiological data will be paramount for determining *Map* transmission events, more so than for faster evolving pathogens.

## Conclusions

This study has clarified the phylogenetic relationships between the different *Map* strain groups that have been reported in the literature. There are two major lineages concordant with the previously described Type S and Type C designations. The Type I and Type III strain groups are subtypes of Type S. The pigmented strains belonged to the Type S lineage but did not comprise a distinct clade in the phylogenetic tree. Although phenotypically distinct from other strains, we could not identify any SNPs, insertions, deletions or the presence or absence of any single gene that could be exclusively associated with pigmented strains. Type B strains are a subtype of Type C and not restricted to *Bison* species. The Indian bison-type is a subtype of Type B. As more isolates are sequenced, more genetic polymorphisms will be identified and the phylogenetic tree will require updating. A central public repository of *Map* genome sequences and corresponding metadata (particularly epidemiological, pathological and virulence data) would greatly facilitate future studies.

With the dataset employed in this study, there was no evidence for strong geographical clustering. The data supports the perception that Type S strains have a preference for sheep and goats suggesting host adaptation may have occurred but this could be biased by the selection of isolates available for study. The human isolates from Crohn’s patients did not comprise a distinct single group but isolates from the same country were often closely related to one another and other *Map* isolates from sheep and cattle from the same country, suggesting livestock as a possible source of transmission.

Overall, the *Map* isolates exhibited restricted genetic diversity and estimates of the substitution rate were less than half a SNP per genome per year, which presents a significant challenge for genotyping and epidemiological tracing. The study highlights the limitations of two commonly used genotyping methods, IS*1311* typing and MIRU-VNTR. IS*1311* typing is used to differentiate between Types S, C and B, but this study suggests that the allelic variation at base pair 223 in IS*1311* used for discrimination occurred after the initial divergence of Type C from Type S strains and does not assign all of the studied strains to the correct lineage. MIRU-VNTR provides more discriminatory power than IS*1311* typing and will differentiate between Type S and Type C but provides limited resolution within these lineages. The polymorphisms detected by MIRU-VNTR do not necessarily accurately reflect the phylogenetic relationships between strains because strains with identical MIRU-VNTR profiles may be distantly related and the data should be treated with caution. WGS provides the ultimate resolution of different isolates and is much more informative than standard genotyping methods. However, WGS alone will not be sufficient for tracing and tracking *Map* infections, yet importantly it can provide a phylogenetic context for affirming epidemiological connections.

WGS of strains passaged *in vitro* has highlighted the mutability of the *Map* genome and how relatively quickly mutations can be selected under different environmental conditions. This emphasizes the need to minimize the subculture of *Map* strains and to use low-passage strains for studies wherever possible.

In summary, this study clarifies the phylogenetic relationships between the previously described *Map* strain groups, and highlights the limitations of current genotyping techniques. WGS of additional isolates will identify further phylogenetic relationships and help to resolve the epidemiology, evolution, population structure and phylogeography of *Map*.

## Additional files

Additional file 1: Table S1. Information on *Map* strains included in this study (XLSX 23 kb) Additional file 2: Figure S1. Maximum likelihood phylogenetic tree of *Map* strains sequenced in this study. The tree was based on the SNPs identified through mapping to *Map* K10 as described in the text and built using RAxML v. 7.0.4 [30] with 100 bootstrap replicates. Branches are annotated with bootstrap values and the tips with the isolate MAPMRI numbers. (PDF 33 kb) Additional file 3: Figure S2. Phylogenetic tree of sequenced and publically available *Map* strains. The data from this study were combined with previously published WGS *Map* data (in red) and the phylogenetic analysis was repeated. The publically available sequences were obtained from [6, 34–37]. At the time of analysis the raw sequencing data was not available for the Camelid strains so short 75 bp paired reads were simulated from the assembled contiguous sequences. (PDF 38 kb) Additional file 4: Figure S3. Comparison between SNP-based phylogeny and MIRU-VNTR and INMV types. The phylogenetic tree presented in Fig. 1 is shown alongside the MIRU-VNTR and INMV types, where colours represent versions of the loci as indicated in the legend. Spaces represent missing data. Dashed lines represent the *Map* strains with identical PFGE/MIRU-VNTR profiles ([2-1]1). (PDF 37 kb)
