# Supplementary figures and images for: Phylogenomic exploration of the relationships between strains of Mycobacterium avium subspecies paratuberculosis
Source: BMC Genomics. 2016 Jan 26;17:79. doi: 10.1186/s12864-015-2234-5 (PMC4729121; doi:10.1186/s12864-015-2234-5)

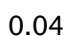

Supplement: Additional file 2: Figure S1. — Maximum likelihood phylogenetic tree of Map strains sequenced in this study. The tree was based on the SNPs identified through mapping to Map K10 as described in the text and built using RAxML v. 7.0.4 [30] with 100 bootstrap replicates. Branches are annotated with bootstrap values and the tips with the isolate MAPMRI numbers. (PDF 33 kb) [file 12864_2015_2234_MOESM2_ESM.pdf]

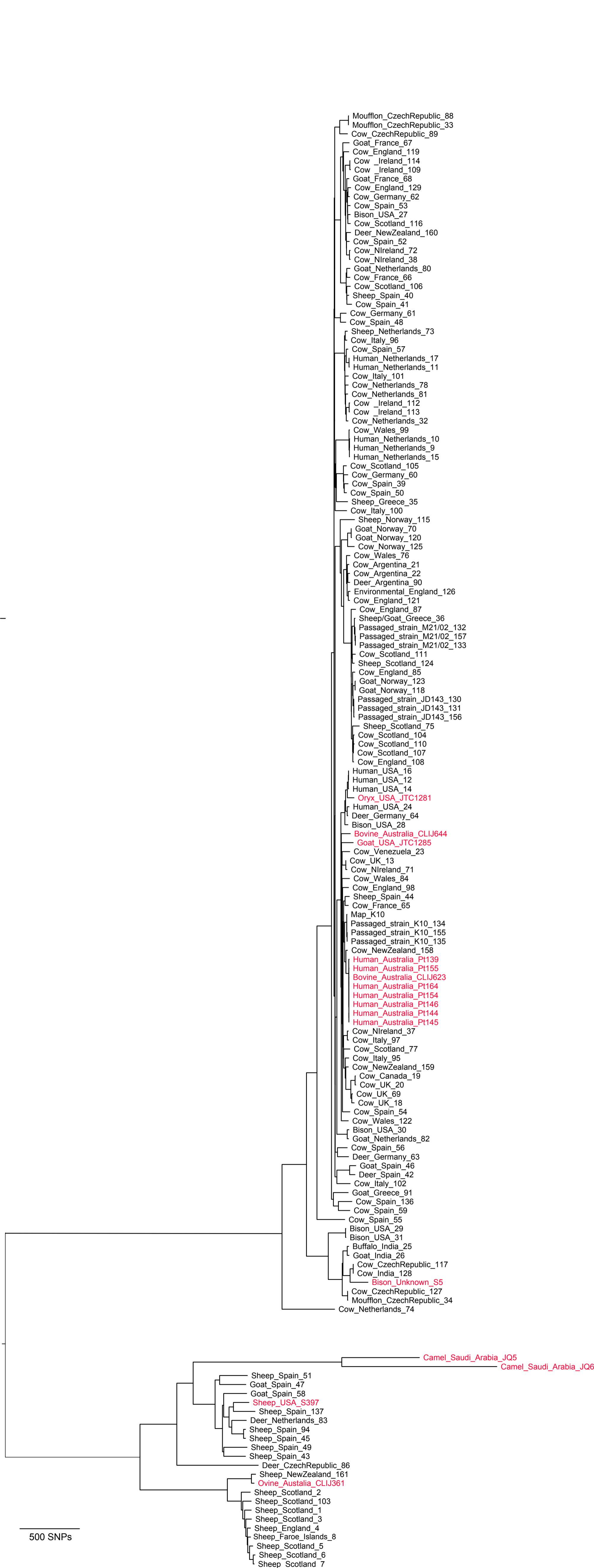

Supplement: Additional file 3: Figure S2. — Phylogenetic tree of sequenced and publically available Map strains. The data from this study were combined with previously published WGS Map data (in red) and the phylogenetic analysis was repeated. The publically available sequences were obtained from [6, 34–37]. At the time of analysis the raw sequencing data was not available for the Camelid strains so short 75 bp paired reads were simulated from the assembled contiguous sequences. (PDF 38 kb) [file 12864_2015_2234_MOESM3_ESM.pdf]

Key (INMV): 1 2 3 6 7 13 17 19 21 25 27 32 33 35 36 68 69 70 71 72 75 78 80 82 84 85 122 126 127 131

Key (MIRU\_292): 2 3 4 5 6 7 8 9 11

Key (MIRU\_x3): 1 2 3

Key (VNTR\_25): 1 2 3 5

Key (VNTR\_47): 2 3 4

Key (VNTR\_3): 1 2

Key (VNTR\_7): 1 2 3 7 8

Key (VNTR\_10): 1 2

Key (VNTR\_32): 6 8 9

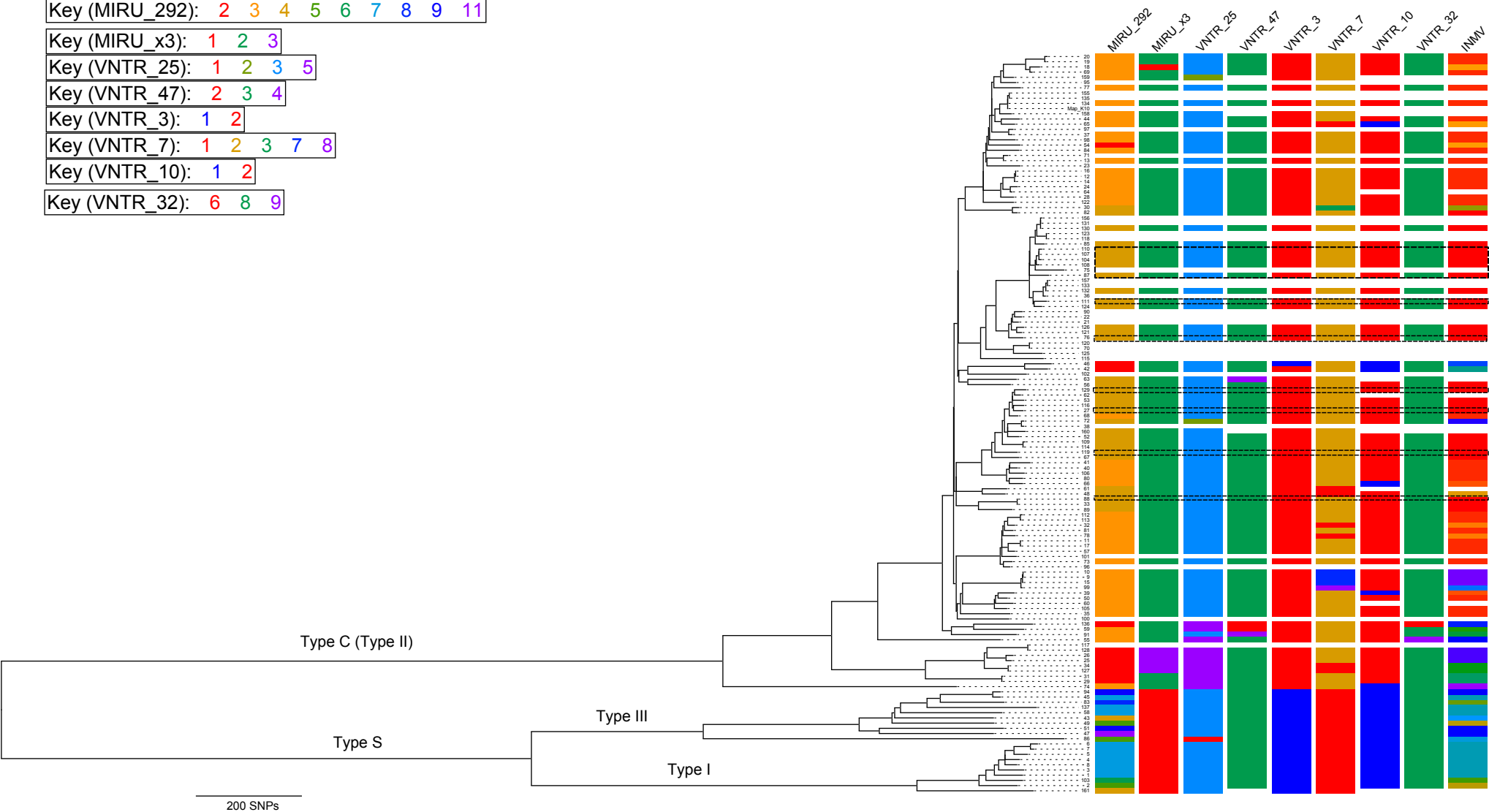

Supplement: Additional file 4: Figure S3. — Comparison between SNP-based phylogeny and MIRU-VNTR and INMV types. The phylogenetic tree presented in Fig. 1 is shown alongside the MIRU-VNTR and INMV types, where colours represent versions of the loci as indicated in the legend. Spaces represent missing data. Dashed lines represent the Map strains with identical PFGE/MIRU-VNTR profiles ([2-1]1). (PDF 37 kb) [file 12864_2015_2234_MOESM4_ESM.pdf]
